# Novel deployment of a covered duodenal stent in open surgery to facilitate closure of a malignant duodenal perforation

**DOI:** 10.1186/1477-7819-7-79

**Published:** 2009-10-27

**Authors:** Philip F Lung, Adrian B Cresswell, Josephine Psaila, Ameet G Patel

**Affiliations:** 1 Department of Hepatobiliary Surgery, King's College Hospital, London, UK

## Abstract

**Background:**

Its a dilemma to attempt a palliative procedure to debulk the tumour and/or prevent future obstructive complications in a locally advanced intra abdominal malignancy.

**Case presentation:**

A 38 year old Vietnamese man presented with a carcinoma of the colon which had invaded the gallbladder and duodenum with a sealed perforation of the second part of the duodenum. Following surgical exploration, it was evident that primary closure of the perforated duodenum was not possible due to the presence of unresectable residual tumour.

**Conclusion:**

We describe a novel technique using a covered duodenal stent deployed at open surgery to aid closure of a malignant duodenal perforation.

## Background

With locally advanced intra-abdominal malignancy the surgeon is faced with the dilemma of attempting a palliative procedure to debulk the tumour and/or prevent future obstructive complications against limiting the impact of any surgical procedure on remaining quality of life. Unfortunately it remains extremely difficult to assess tumours which are adherent to local structures and the decision must be made whether to continue anatomical dissection or to leave the main tumour bulk in-situ and perform a simple bypass procedure.

## Case presentation

A 38 year old Vietnamese man was admitted with a 10 month history of epigastric pain, fatigue, 10 kg weight loss and recent onset jaundice. He had no other significant medical history. Clinical examination demonstrated anaemia and a tender mass in the right upper quadrant of the abdomen. A computerised tomography (CT) scan of the abdomen revealed a 7 × 5 cm thick-walled, complex mass adjacent to the second part of the duodenum, which contained fluid and air and abutted the hepatic flexure of the colon. The working diagnosis was a collection secondary to a colonic perforation and he was treated with intravenous antibiotics. He improved with conservative management and was discharged a month later for outpatient colonoscopy. The colonoscopy revealed a lesion in the transverse colon, histology of which showed a mucinous adenocarcinoma.

He subsequently returned to the Accident and Emergency Department following an upper gastrointestinal bleed. On his second admission, a repeat CT scan again suggested localised colonic perforation with formation of an abscess adjacent to the duodenum, along with thickening of the ascending colon, predominantly centred around the hepatic flexure. Given the clinical presentation and diagnostic uncertainty a diagnostic laparoscopy was performed which revealed a large perforated tumour at the hepatic flexure with ascites and peritoneal tumour nodules. A laparotomy was performed via a transverse incision and following mobilisation of the hepatic flexure, a colonic tumour was found to have invaded the gallbladder and duodenum with an abscess cavity anterior to the second part of the duodenum. At the base of the abscess cavity a large hole was apparent in the second part of the duodenum with malignant tumour invading the duodenum. Given the size of the defect (5 cm × 2 cm) and the presence of tumour it was not possible to resect and form a primary closure of the duodenum. The presence of metastatic spread precluded a curative resection by pancreatoduodenectomy.

A right hemicolectomy was performed to debulk the tumour and an ileotransverse anastamosis formed. Due to the extent of the disease and associated abscess the anterior wall of the duodenum came away with the colon during this manoeuvre. A retrograde cholecystectomy was carried out to resect the residual tumour invading the gall bladder. The ampulla was identified with the aid of a transcystic catheter. An 82 mm expandable covered duodenal stent with a diameter of 18 mm (Hanarostent, duodenal/pyloric, M.I. Tech Co. Ltd, Seoul, N. Korea) was manually inserted through the duodenal perforation into the proximal duodenum with the distal end of the stent inserted into D2. A small opening into the side wall of the stent was made prior to the positioning of the stent to accommodate the ampulla and thus facilitate drainage of bile within the stent. A per-operative cholangiogram confirmed free flow of contrast in the duodenum. The residual duodenal wall was closed over the stent and following antrectomy a gastrojejunostomy was formed to bypass the duodenum (Bilroth II reconstruction).

The patient was discharged 7 weeks later following a prolonged, but otherwise uncomplicated recovery. He subsequently underwent a palliative course of chemotherapy survived for a further 18 months without gastrointestinal symptoms, before succumbing to his disease.

## Discussion

The major risk with attempting palliative resection is the feasibility of reconstructing anatomy that has been altered by the malignant process. In the case described separation of the main tumour bulk from the duodenum left a perforation involving most of the anterior surface of the second part of the duodenum. Significantly sized malignant perforations of the duodenum are unlikely to heal following simple primary closure and are at risk of resulting in high output fistulas or high level GI tract obstruction due to disease progression. Simple duodenal closure and duodenal bypass in this case would not have been sufficient to safely defunction the perforation.

Several techniques have been described for closure of large duodenal perforations, such as duodenojejunostomy, serosal and mucosal pedical patching [1-3] without recourse to pancreatoduodenectomy. These procedures were mainly described for patients who sustained severe duodenal trauma, rather than in the context of malignant perforation. All of these techniques would have been at high risk of anastomotic leakage and/or subsequent obstruction in this patient due to progression of the underlying residual disease.

Covered self-expanding metallic stents have been used to treat oesophageal leaks and fistulas with clinical success ranging from 67-100%, with recurrent fistulas or leaks in 8-20% of patients [4-7]. Placement of duodenal stents in malignancy has so far been described as an endoscopic procedure [8,9] to relieve obstruction and not to aid in sealing a duodenal perforation from tumour invasion. These stents are effective in the palliation of symptoms of gastric outlet obstruction in patients with a relatively short life expectancy. However, performing a gastrojejunostomy may be considered more appropriate for those with a more favourable prognosis [10].

## Conclusion

We believe that in this case, the novel deployment of a covered duodenal stent during an open operation negated the requirement for a major pancreatobiliary resection and permitted safe primary closure of the malignant perforation. This allowed the early use of palliative chemotherapy and, in conjunction with tumour debulking, may have resulted in a significant gain in symptom free survival.

## Competing interests

The authors declare that they have no competing interests.

## Authors' contributions

PFL helped in preparation of the manuscript and literature search. ABC helped in preparation of the manuscript and literature search. JP helped in preparation of the manuscript, literature search and editing the manuscript for its final content. AGP helped in preparation of the manuscript, literature search and editing the manuscript for its final content. All authors read and approved the final manuscript.

## Consent

The publication of this case was approved by the National Research Ethics Service, King College Hospital Research Ethics committee, as the consent from the patient and next of kin was not possible. The copy of ethical committee approval is available with chief editor.
